# Changes in management of idiopathic pulmonary fibrosis: impact on disease severity and mortality

**DOI:** 10.1080/20018525.2020.1807682

**Published:** 2020-08-12

**Authors:** Charlotte Hyldgaard, Janne Møller, Elisabeth Bendstrup

**Affiliations:** a Diagnostic Center, University Research Clinic for Innovative Patient Pathways, Silkeborg Regional Hospital, Silkeborg, Denmark; b Center for Rare Lung Diseases, Department of Respiratory Diseases and Allergy, Aarhus University Hospital, Aarhus, Denmark

**Keywords:** Idiopathic pulmonary fibrosis, mortality, antifibrotic therapy, interstitial lung disease

## Abstract

**Background:**

Idiopathic pulmonary fibrosis (IPF) is a serious interstitial lung disease (ILD) with a median survival of 3-5 years. The aim of the present study was to evaluate disease severity and survival in patients diagnosed with IPF in the era of antifibrotic therapies compared with an earlier IPF cohort.

**Methods:**

We identified all patients with fibrotic ILD in the hospital electronic case record system between 2011 and 2016, and reviewed each case in order to identify incident patients with IPF. We used the GAP-index to compare disease severity and mortality to previous findings in patients with IPF diagnosed at our center between 2003 and 2009.

**Results:**

260 patients were diagnosed with IPF between 2011 and 2016. Mean age was 72.6 years, 79% were male, mean forced vital capacity (FVC) was 80%, and mean diffusing capacity for carbon monoxide (DLco) was 44%. Age, FVC and DLco were significant predictors of mortality, but the presence of a typical usual interstitial pneumonia pattern on HRCT was not. Eighty percent of patients in GAP stage I received antifibrotic therapy, 73% in GAP stage II, and 29% in GAP stage III.

The median survival was four years in the 2011-2016 cohort compared with three years in the 2003-2009 cohort. The distribution of patients between GAP stages was unchanged in 2011-2016 compared with 2003-2009, (stage I 34% vs. 32%, stage II 49% vs. 48% and stage III 20% vs. 16%). One-year mortality was 13% in 2011-2016 and 26% in 2003-2009. In severe disease (GAP stage III), one-year mortality was 26% and 54%, respectively, (p=0.019).

**Conclusion:**

Short-term mortality was significantly lower in the 2011-2016 cohort compared with 2003-2009. This improvement may be linked to changes in treatment strategies towards limited use of corticosteroids. Although early diagnosis of IPF still needs increased focus, the improvement is encouraging.

## Background

Idiopathic pulmonary fibrosis (IPF) is a serious lung disease, which mainly affects male patients in their sixties and seventies [1]. The disease is usually progressive, and most centers report a median survival of three to five years [2,3]. Since 2011, antifibrotic therapy has been available with the potential of slowing disease progression and increasing survival [4–6].

We have previously described a cohort of patients with IPF diagnosed and followed at our center between 2003 and 2009 [7]. The aim of the present study was:

1. To investigate disease severity at the time of referral in a recent cohort of patients with IPF diagnosed and followed in the era of antifibrotic therapies.
2. To characterize and compare clinical characteristics, treatment patterns and mortality in the two cohorts.

We hypothesized that better diagnostic strategies and increased awareness of IPF owing to the emergence of evidence-based therapies would result in earlier diagnosis and improved survival.

## Methods

In this retrospective cohort study, we included all patients diagnosed with IPF between September 2011 and August 2016 at Center for Rare Lung Diseases, Department of Respiratory Diseases and Allergy, Aarhus University Hospital, Denmark, which is the referral center for the Central Denmark Region with a population of 1.3 million (2018).

IPF was diagnosed according to the 2011 ATS/ERS/JRS/ALAT criteria [1]. For the purpose of the study, high resolution computed tomography (HRCT) scans performed at the time of diagnosis were re-evaluated according to the 2018 Fleischner Society criteria for IPF [8]. We identified all patients with the ICD-10 diagnostic code J84X in the hospital’s electronic case record system in order to identify patients with IPF and to ensure inclusion of patients diagnosed with IPF in case the referral diagnosis had not been changed to J84.1A. Data collection was based on the electronic case records. Patients were followed from the time of the first visit to the center, and follow-up was carried out until May 2018. Vital status was assessed through the hospital electronic files that contain continuously updated information based on the Danish civil registration system. This approach ensured complete follow up with respect to mortality.

Data are presented as mean ± SD if continuous or as frequencies if categorical. Survival was evaluated using the Kaplan-Meier method and differences in survival curves were evaluated using the log-rank test. Cox proportional hazards regression was used to estimate hazard rate ratios for death and corresponding 95% confidence intervals. The GAP index [9] was used to categorise patients into three prognostic stages based on gender, age, forced vital capacity (FVC), and diffusing capacity of the lung for carbon monoxide (DLco).”

All analyses were performed using STATA statistical software (version 12.1; StataCorp, College Station, Texas, USA).

The study was approved by The Danish Patient Safety Authority (record number 3–3013-1993/1).

## Results

Two hundred and sixty patients were included in the study, corresponding to an incidence of 3.3 per 100,000 population in the Central Denmark Region. One-hundred forty-two patients (55%) died during the study period, and median survival was four years. Mean age at the time of diagnosis was 72.6 years and 79% of the patients were male. At the time of diagnosis, 55% of the patients had a typical UIP pattern on HRCT. Overall, 84 patients (32%) had a biopsy, either video-assisted thoracoscopic surgery (VATS) (63/84, 75%) or cryobiopsy (21/84, 25%). The cryobiopsy technique was introduced at the center in 2016. Seventy-five patients with a probable UIP pattern on HRCT underwent a biopsy (54 VATS and 21 cryobiopsies). Forty-one patients (16%) had a probable UIP pattern on HRCT and were assigned a working diagnosis of IPF without a biopsy according to the 2018 Fleischner Society criteria for IPF [8].

Patients with a probable UIP pattern, who underwent a biopsy, had mean FVC of 85% predicted and mean DLco of 53% predicted. Patients with a probable UIP pattern on HRCT, who were diagnosed without a biopsi, had mean FVC of 75% predicted and mean DLco of 41% predicted. Bronchoalveolar lavage (BAL) was performed in 72% of the patients. BAL differential count was available in 182 of the 186 cases. The mean BAL lymphocyte count was 8%; neutrophil count 10%; and macrophage count 75%. Demographic characteristics are shown in Table 1.

**Table 1.** Demographic characteristics in the 2011–2016 and the 2003–2009 IPF cohorts.

	2011–2016 IPF cohort n = 260	2003–2009 IPF cohort n = 121
Male gender, n (%)	205 (79)	93 (77)
Mean age, years (SD) Median age, years (interquartile range)	72.6 (8.4) 73.1 (67.8–78.4)	67.4 (8.4) 68.2 (61.2–74.0)
Smokers, n (%) Current, n (%) Previous, n (%) Never, n (%) Unknown, n (%)	190 (73) 15 (6) 175 (67) 62 (24) 8 (3)	98 (81) 16 (13) 82 (68) 23 (19) 0 (0)
Pack-years (SD)	28 (20)	29 (17)
HRCT Typical UIP pattern, n (%) Biopsy Probable UIP pattern, n (%) Biopsy No biopsy, clinical diagnosis Indeterminate for UIP, n (%)	260 (100) 144 (55) 9 (6) 116 (45) 75 (65) 41 (35) 0 (0)	121 (100) 60 (50) 14 (23) 61 (50) 38 (62)) 23 (38) 0 (0)
Biopsy, n (%) VATS, n (%) Cryobiopsy, n (%)	84 (32) 63 (75) 21 (25)	52 (43) 52 (100) 0
Bronchoalveolar lavage (%)	186 (72)	93 (77)
FVC % predicted (SD)	80 (22)	72 (21)
DLco % predicted (SD)	44 (15)	42 (16)
Antifibrotic therapy, n (% of entire cohort) Nintedanib, n (% of treated patients)* Pirfenidon, n (% of treated patients)* Both (not concomitantly), n (% of treated patients)	175 (67) 36 (21) 125 (71) 14 (8)	Antifibrotic therapy not available
Mean observation time (years) (SD)	2.7 (1.7)	1.96 (1.6)

SD: standard deviation, HRCT: high-resolution computed tomography, UIP: usual interstitial pneumonia, VATS: video-assisted thoracoscopic surgery, FEV1: forced expiratory volume in one second, FVC: forced vital capacity, TLC: total lung capacity, DLco: diffusion capacity of carbon monoxide.

*Pirfenidone was approved in Denmark in 2011 and nintedanib in 2015

In a univariate regression model, age, FVC, and DLco were significant predictors of mortality, but HRCT pattern was not. In a multivariate model including these parameters, the same pattern was seen: age, FVC, and DLco remained significant while the presence of a UIP pattern did not predict a worse outcome (Tables 2 and 3).

**Table 2.** Univariate analyses for predictors of mortality in the 2011–2016 cohort.

	Hazard ratio (95% CI)	p
Gender	1.25 (0.75; 2.11)	0.40
Age	1.05 (1.02; 1.08)	<0.001
Smoking history	0.86 (0.61; 1.22)	0.40
FVC	0.98 (0.97; 0.99)	<0.001
DLco	0.95 (0.94; 0.97)	<0.001
HRCT pattern	1.39 (0.95; 2.02)	0.09

**Table 3.** Multivariate model for predictors of mortality in the 2011–2016 cohort.

	Hazard ratio (95% CI)	p
Gender	0.90 (0.57;1.41)	0.646
Age	1.05 (1.02; 1.08)	<0.001
Smoking history	0.99 (0.72; 1.38)	0.970
FVC	0.97 (0.96; 0.98)	<0.001
DLco	0.95 (0.93;0.96)	<0.001
HRCT pattern	0.92 (0.65; 1.31)	0.638

Using the GAP score as a tool for prognostic stratification, 34% of the patients were in stage I, 49% were in stage II, and 16.0% were in stage III (Table 4).

**Table 4.** Clinical characteristics for GAP stages I–III in the 2003–2009 cohort, (n = 115) and the 2011–2016 cohort, (n = 257).

	Gender	Age	FVC	DLco
**GAP I** 2003–2009 (37/115, 32%) 2011–2016 (88/257, 34%)	67% male 61% male	61.8 years 68.8 years	82.3% pred. 93.7% pred.	52.9% pred. 56.0% pred.
**GAP II** 2003–2009 (55/115, 48%) 2011–2016 (127/257, 49%)	80% male 87% male	68.8 years 73.8 years	73.1% pred. 77.8% pred.	38.1% pred. 41.0% pred.
**GAP III** 2003–2009 (23/115, 20%) 2011–2016 (42/257, 16%)	78% male 93% male	71.6 years 76.0 years	46.0% pred. 55.2% pred.	26.9% pred. 29.0% pred.
0 points 1 points 2 points 3 points	female male	<60 years 61–65 years >65 years	>75% pred. 50–75% pred. <50% pred.	>55% pred. 36–55% pred. <35% pred. unable to perform

Three patients in the 2011–2016 cohort (1%) and six patients (5%) in the 2003–2009 cohort had insufficient pulmonary function data for GAP staging.

### Comparison of the 2011-2016 cohort and the 2003-2009 cohort

**Figure 1.**
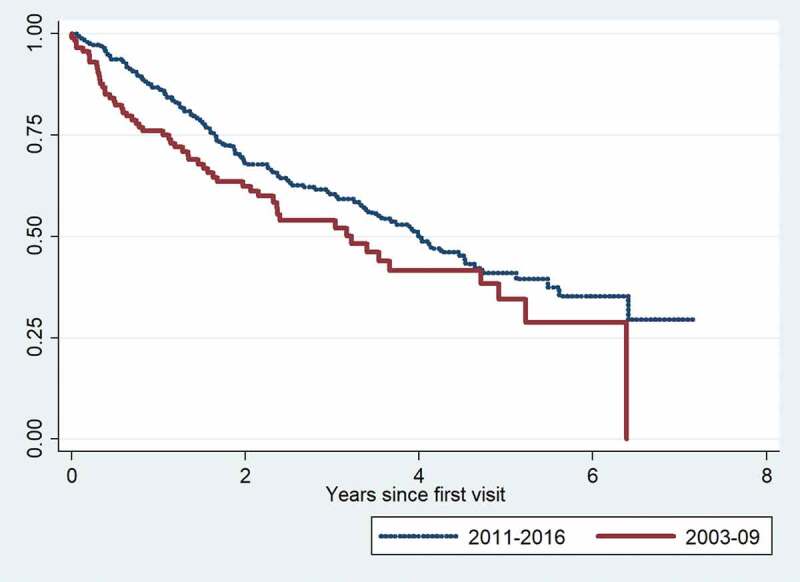
Kaplan-Meier mortality curves for the 2003–2009 and 2011–2016 cohort.

We compared the 2011–2016 cohort to a cohort of patients with IPF diagnosed and treated at the center between 2003 and 2009. The distribution of gender, age and pulmonary function levels among the three GAP stages for each cohort are shown in Table 4. Median survival was 4.0 years in the 2011–2016 cohort and 3.2 years in the 2003–2009 cohort. The overall survival was significantly better in the 2011–2016 cohort (p = 0.046). Survival curves for the two cohorts are shown in Figure 1.

p = 0.046

**Figure 2.**
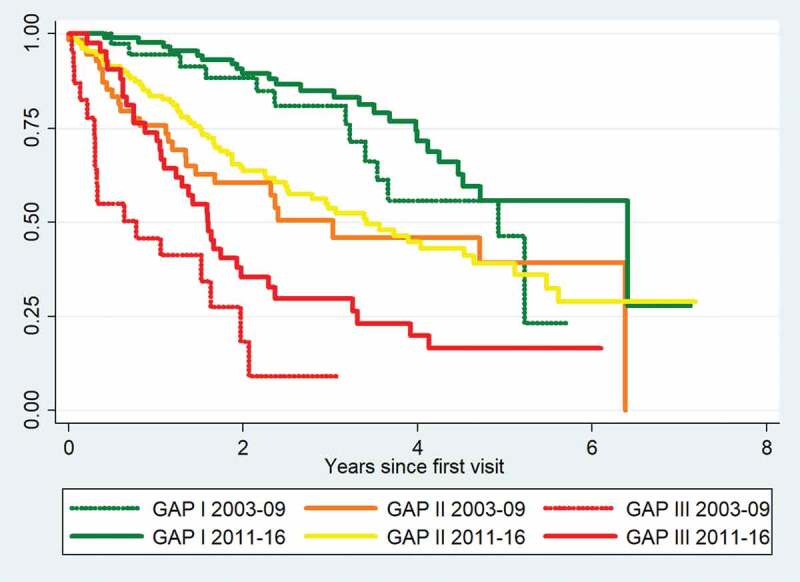
Kaplan-Meier mortality curves for GAP stages I–III in the 2003–2009 and 2011–2016 cohorts.

Among patients with severe disease at the time of referral (GAP stage III), one year mortality declined from 54.2% in the 2003–2009 cohort to 26.2% in the 2011–2016 cohort (Table 5). We performed a comparison of the survival curves for patients in GAP stage I in the 2011–2016 cohort and the 2003–2009 cohort and found no difference in survival (p = 0.15). The same was observed for GAP stage II in the two cohorts (p = 0.46). For GAP stage III, the survival was significantly higher among patients in the 2011–2016 cohort (p = 0.019). Survival curves for each GAP stage in the two cohorts are shown in Figure 2.

**Table 5.** One-year and three-year mortality by GAP stages I–III for the 2003–2009 and 2011–2016 cohorts.

	One-year mortality	Three-year mortality
GAP stage I 2003–2009 2011-2016	5.5% 2.3%	19.1% 15.1%
GAP stage II 2003–2009 2011-2016	24.5% 16.5%	49.5% 46.3%
GAP stage III 2003–2009 2011-2016	54.2% 26.2%	81.8% 70.3%

Sixty-seven percent of patients in the 2011–2016 cohort received antifibrotic therapy. Criteria for reimbursement were FVC>50% and DLco>30%. In GAP stage I, 70/88 patients (80%) received antifibrotic therapy, in GAP stage II, 93/127 (73%) and in GAP stage III, 12/42 (29%). Twelve of 18 untreated patients in GAP stage I did not wish to receive antifibrotic therapy. The remaining six patients had comorbidities that interfered with therapy or were unable to stop smoking, which was a prerequisite for treatment with pirfenidone. In GAP stage II, 14 patients had pulmonary function below the limits; five patients did not wish to receive antifibrotic therapy; eight patients received the IPF diagnosis in retrospect and therefore did not receive antifibrotic therapy; and seven patients had other reasons. In GAP stage III, 38/42 patients had pulmonary function below the limits; two patients were not treated because of high age; and two patients were diagnosed with fibrotic NSIP initially, but IPF after re-evaluation.

In the 2003–2009 cohort, 85% of the patients received corticosteroids, either daily Prednisolone (75%) or high-dose methylprednisolone pulse therapy (53%) or both, whereas 62% of the patients received azathioprine [10]. In the 2011–2016 cohort, oral corticosteroids or other immunosuppressants as IPF-directed therapy was not used except for low dose corticosteroids as part of a palliative strategy in very severe disease and high-dose methylprednisolone for acute exacerbations.

## Discussion

The number of incident patients with IPF more than doubled in the 2011–2016 cohort compared with 2003–2009. Patients in the 2011–2016 cohort were older at the time of referral (mean age 72.6 years in 2011–2016 and 67.4 years in 2003–2009), mean FVC was slightly better (80% vs. 72%), but DLco was similar (44% vs. 42%). However, the distribution of patients according to GAP stages remained unchanged. Median survival increased from three to four years, although the patients on average were five years older at the time of diagnosis. The most striking finding was the decrease in one-year mortality from 26% to 13% overall. Among patients in GAP stage III, one-year mortality decreased from 54% to 26%.

The treatment recommendations at IPF centers worldwide changed in 2011 after the interim results of the Panther trial showed excess mortality among patients treated with high-dose Prednisolone and Azathioprine [11]. These results caused a prompt change of practice at our center, and this may explain the decrease in short-term mortality for patients with very severe disease.

The increase in the number of referrals likely reflects increased awareness of fibrotic ILD among pulmonologists and radiologists in our region, which may be driven by the emergence of evidence based therapies. Furthermore, the use of a provisional high-confidence diagnosis or ‘working diagnosis’ of IPF has been introduced, usually for patients with advanced disease in whom a histopathological confirmation of the diagnosis is not possible. This group of patients mainly have a probable UIP pattern on HRCT, and they have the same disease course and response to antifibrotic therapy as those with a definite UIP pattern or a histopathological diagnosis confirming IPF [4]. Previous reports have pointed towards a ‘wait and see’ approach to therapy in mild disease [12,13]. This did not seem to be the case in our study with 80% of patient in GAP stage I and 73% of patients in GAP stage II on antifibrotic therapy. Other Nordic countries have divergent approaches to antifibrotic therapies with large differences in the proportion of patients who receive antifibrotic therapy. In Finland, 26% of the patients were on therapy for at least six months compared to 69% in Sweden [14]. The proportion of patients who received antifibrotic therapy was also higher in our cohort than in other real life cohorts (23% in the Australian cohort and 44% in the German cohort, but similar to the findings in a Czech cohort (63% treated patients) [2,3,15].)

We found comparable mortality between patients who had a definite UIP pattern on HRCT and those who had probable UIP. Previous studies have also reported that a definite UIP pattern on HRCT is not an independent predictor of mortality [16–20]. Some patients in our study were initially diagnosed as having fibrotic NSIP based on HRCT pattern and no biopsy, and these patients did not receive antifibrotic therapy. After re-evaluation, they were reclassified as having IPF in accordance with the progress that has been made in the understanding of the disease behaviour.

Several reports of other ‘real life’ cohorts have been published. Some of these include prevalent as well as incident patients. Many similarities exist between our cohort and the Australian IPF cohort [2] with regards to pulmonary function (FVC 81% and DLco 48%), median survival of approximately four years, and more than 70% of the patients having a smoking history. Some similarities are also seen with a recent report from the Finnish IPF registry [21]: age at the time of diagnosis and mortality are comparable, although the Finnish cohort had fewer smokers; a higher proportion of female patients; and the patients had more preserved pulmonary function at the time of diagnosis. Thus, a higher proportion of the Finnish patients were in GAP stage I at the time of diagnosis (54% in Finland vs 34% in Aarhus). Survival in GAP stage I and II were higher in the Finnish cohort, but similar in GAP stage III. Although our study reveals some improvement in outcome for patients with IPF, especially regarding short-term survival, the distribution of patients between GAP stages remains unchanged. This finding underlines the persistent need to improve identification of fibrotic lung disease at an earlier stage.

Recent studies show that misdiagnoses and delay are still major problems in IPF [22,23].

Patients in GAP stage III were of course older and had more severely impaired pulmonary function, since age and pulmonary function are part of the GAP index. Only 29% of our patients in GAP stage III received antifibrotic therapy. The main reason for abstaining from therapy in this group was low lung function. Physicians and/or patients may be concerned about potential adverse effects or lack of treatment effect in severe disease. However, a recent study reported that the adverse event profile was similar between age groups, but a greater proportion of patients aged ≥75 than patients aged <75 discontinued therapy due to adverse events [24]. Two recent studies have shown that antifibrotic therapy with pirfenidone results in similar rate of lung function decline and similar safety profile in patients with more advanced versus less advanced IPF. One study reported results from RECAP, the extension study from the phase 3 trials of pirfenidone in patients with IPF, ASCEND and CAPACITY, [25] and another study reported the outcomes of participants in the ASCEND and CAPACITY trials whose pulmonary function for different reasons were below the screening criteria [26]. Compared with placebo, patients with pulmonary function below the screening criteria who received pirfenidone had significantly reduced mortality and significantly less deterioration in lung function, exercise capacity and dyspnoea. A recent study have reported a similar effect of nintedanib on FVC decline in patients with IPF and more versus less severe impairment in DLco [27].

These emerging reports of treatment response and tolerability among patients with advanced disease are promising, and they may lead to inclusion of patients with more advanced disease in future IPF clinical trials.

The study is limited by the retrospective design and inclusion of patients from a single referral center. However, using the same approach to patient identification and cohort description in two distinct periods at one center with unchanged referral procedure allows the comparison of clinical characteristics among patients with IPF.

Our study strongly suggests that patients with very severe IPF do better after immunosuppressive therapies have become obsolete, but the optimal use of antifibrotic therapies in severe IPF remains to be clarified.

## Conclusion

Disease severity at the time of IPF diagnosis in our study was unchanged between 2003–2009 and 2011–2016, which contradicts our hypothesis of earlier diagnosis. Nevertheless, we saw a significant mortality decline in 2011–2016, although the patients were older. The mortality decline was driven mainly by the decline in short-term mortality among patients with severe disease, and may be linked to the changes in treatment strategies towards limited use of corticosteroids as well as the introduction of antifibrotic therapy..

## References

[1] Raghu G, Collard HR, Egan JJ, et al. An official ATS/ERS/JRS/ALAT statement: idiopathic pulmonary fibrosis: evidence-based guidelines for diagnosis and management. Am J Respir Crit Care Med. 2011 3 15;183(6):788–7. 21471066 10.1164/rccm.2009-040GL PMC5450933

[2] Jo HE, Glaspole I, Grainge C, et al. Baseline characteristics of idiopathic pulmonary fibrosis: analysis from the Australian Idiopathic Pulmonary Fibrosis Registry. Eur Respir J. 2017 2 23;49(2). DOI:10.1183/13993003.01592-2016 28232409

[3] Behr J, Kreuter M, Hoeper MM, et al. Management of patients with idiopathic pulmonary fibrosis in clinical practice: the INSIGHTS-IPF registry. Eur Respir J. 2015 7;46(1):186–196. 25837040 10.1183/09031936.00217614 PMC4486374

[4] Richeldi L, Du Bois RM, Raghu G, et al. Efficacy and safety of nintedanib in idiopathic pulmonary fibrosis. N Engl J Med. 2014 5 29;370(22):2071–2082. 24836310 10.1056/NEJMoa1402584

[5] King TE Jr, Bradford WZ, Castro-Bernardini S, et al. A phase 3 trial of pirfenidone in patients with idiopathic pulmonary fibrosis. N Engl J Med. 2014 5 29;370(22):2083–2092. 24836312 10.1056/NEJMoa1402582

[6] Noble PW, Albera C, Bradford WZ, et al. Pirfenidone in patients with idiopathic pulmonary fibrosis (CAPACITY): two randomised trials. Lancet. 2011 5 21;377(9779):1760–1769. 21571362 10.1016/S0140-6736(11)60405-4

[7] Hyldgaard C, Hilberg O, Muller A, et al. A cohort study of interstitial lung diseases in central Denmark. Respir Med. 2014 5;108(5):793–799. 24636811 10.1016/j.rmed.2013.09.002

[8] Lynch DA, Sverzellati N, Travis WD, et al. Diagnostic criteria for idiopathic pulmonary fibrosis: a Fleischner society white paper. Lancet Respir Med. 2018 2;6(2):138–153. 29154106 10.1016/S2213-2600(17)30433-2

[9] Ley B, Ryerson CJ, Vittinghoff E, et al. A multidimensional index and staging system for idiopathic pulmonary fibrosis. Ann Intern Med. 2012 5 15;156(10):684–691. 22586007 10.7326/0003-4819-156-10-201205150-00004

[10] Hyldgaard C, Hilberg O, Bendstrup E. How does comorbidity influence survival in idiopathic pulmonary fibrosis? Respir Med. 2014 4;108(4):647–653. 24529739 10.1016/j.rmed.2014.01.008

[11] Raghu G, Anstrom KJ, King TE, et al., Idiopathic Pulmonary Fibrosis Clinical Research Network. Prednisone, azathioprine, and N-acetylcysteine for pulmonary fibrosis. N Engl J Med. 2012 5 24;366(21):1968–1977. 22607134 10.1056/NEJMoa1113354 PMC3422642

[12] Maher TM, Strek ME. Antifibrotic therapy for idiopathic pulmonary fibrosis: time to treat. Respir Res. 2019 9 6;20(1):1–4. 31492155 10.1186/s12931-019-1161-4 PMC6731623

[13] Maher TM, Molina-Molina M, Russell AM, et al. Unmet needs in the treatment of idiopathic pulmonary fibrosis-insights from patient chart review in five European countries. BMC Pulm Med. 2017 9 15;17(1):124. 28915874 10.1186/s12890-017-0468-5 PMC5602932

[14] Pesonen I, Carlson L, Murgia N, et al. Delay and inequalities in the treatment of idiopathic pulmonary fibrosis: the case of two Nordic countries. Multidiscip Respir Med. 2018 5 14;13:1–6. 29785264 10.1186/s40248-018-0126-7 PMC5950183

[15] Zurkova M, Kriegova E, Kolek V, et al. Effect of pirfenidone on lung function decline and survival: 5-yr experience from a real-life IPF cohort from the Czech EMPIRE registry. Respir Res. 2019 1 21;20(1):16–019-0977-2. 10.1186/s12931-019-0977-2 PMC6341650 30665416

[16] Raghu G, Wells AU, Nicholson AG, et al. Effect of nintedanib in subgroups of idiopathic pulmonary fibrosis by diagnostic criteria. Am J Respir Crit Care Med. 2017 1 1;195(1):78–85. 27331880 10.1164/rccm.201602-0402OC PMC5214917

[17] Yamauchi H, Bando M, Baba T, et al. Clinical course and changes in high-resolution computed tomography findings in patients with idiopathic pulmonary fibrosis without honeycombing. PloS One. 2016 11 9;11(11):e0166168. 27829068 10.1371/journal.pone.0166168 PMC5102464

[18] Gruden JF, Panse PM, Leslie KO, et al. UIP diagnosed at surgical lung biopsy, 2000-2009: HRCT patterns and proposed classification system. AJR Am J Roentgenol. 2013 5;200(5):W458–67. 23617514 10.2214/AJR.12.9437

[19] Lee JW, Shehu E, Gjonbrataj J, et al. Clinical findings and outcomes in patients with possible usual interstitial pneumonia. Respir Med. 2015 4;109(4):510–516. 25736347 10.1016/j.rmed.2015.02.008

[20] Sumikawa H, Johkoh T, Colby TV, et al. Computed tomography findings in pathological usual interstitial pneumonia: relationship to survival. Am J Respir Crit Care Med. 2008 2 15;177(4):433–439. 17975197 10.1164/rccm.200611-1696OC

[21] Kaunisto J, Salomaa ER, Hodgson U, et al. Demographics and survival of patients with idiopathic pulmonary fibrosis in the Finnish IPF registry. ERJ Open Res. 2019 7 8;5(3). DOI:10.1183/23120541.00170-2018 PMC6612605 31304177

[22] Cosgrove GP, Bianchi P, Danese S, et al. Barriers to timely diagnosis of interstitial lung disease in the real world: the INTENSITY survey. BMC Pulm Med. 2018 1 17;18(1):9–017-0560-x. 10.1186/s12890-017-0560-x PMC5773175 29343236

[23] Hoyer N, Prior TS, Bendstrup E, et al. Risk factors for diagnostic delay in idiopathic pulmonary fibrosis. Respir Res. 2019 5 24;20(1):103. 31126287 10.1186/s12931-019-1076-0 PMC6534848

[24] Bendstrup E, Bonella F, Bargagli E, et al. Efficacy and safety of nintedanib in the elderly patient with IPF. Abstract PA1353 presented at the European Respiratory Society Congress, Madrid, Spain. 2019.

[25] Costabel U, Albera C, Glassberg MK, et al. Effect of pirfenidone in patients with more advanced idiopathic pulmonary fibrosis. Respir Res. 2019 3 12;20(1):1–4. 30866942 10.1186/s12931-019-1021-2 PMC6416878

[26] Nathan SD, Costabel U, Albera C, et al. Pirfenidone in patients with idiopathic pulmonary fibrosis and more advanced lung function impairment. Respir Med. 2019;153:44–51. 31153107 10.1016/j.rmed.2019.04.016

[27] Richeldi L, Kolb M, Jouneau S, et al. Efficacy and safety of nintedanib in patients with advanced idiopathic pulmonary fibrosis. BMC Pulm Med. 2020 1 8;20(1):3. 31914963 10.1186/s12890-019-1030-4 PMC6951000

